# Molecular detection of *Enterocytozoon bieneusi* in farm-raised pigs in Hainan Province, China: infection rates, genotype distributions, and zoonotic potential

**DOI:** 10.1051/parasite/2020009

**Published:** 2020-03-04

**Authors:** Huan-Huan Zhou, Xin-Li Zheng, Tian-Ming Ma, Meng Qi, Jing-Guo Zhou, Hai-Ju Liu, Gang Lu, Wei Zhao

**Affiliations:** 1 Department of Pathogenic Biology, Hainan Medical University Haikou 571199 Hainan PR China; 2 Hainan Medical University-The University of Hong Kong Joint Laboratory of Tropical Infectious Diseases, Hainan Medical University Haikou 571199 Hainan PR China; 3 Key Laboratory of Tropical Translational Medicine of the Ministry of Education, Hainan Medical University 571199 Haikou PR China; 4 College of Animal Sciences, Tarim University Alar 843300 Xinjiang PR China; 5 Institute of Animal Science and Veterinary Medicine, Hainan Academy of Agricultural Sciences 571100 Haikou PR China; 6 Department of Parasitology, Wenzhou Medical University Wenzhou 325035 Zhejiang Province PR China

**Keywords:** *Enterocytozoon bieneusi*, Genotype, ITS region, Pigs

## Abstract

*Enterocytozoon bieneusi* is a zoonotic fungal pathogen with a high degree of host diversity that can parasitize many animals, including humans. Pigs may play an important role in the epidemiology of *E. bieneusi* as reservoir hosts. Nevertheless, the genotypes of *E. bieneusi* in pigs in China remain poorly understood. The aim of this study was to determine the prevalence of *E. bieneusi* infection amongst pigs raised on farms from four cities of Hainan Province, using nested polymerase chain reaction (PCR) of the partial small subunit of the ribosomal RNA gene, and to identify genotypes of *E. bieneusi* isolates based on sequence analysis of the ribosomal internal transcribed spacer (ITS) region. Among 188 stool samples, *E. bieneusi* was detected in 46.8% (88/188). Eight genotypes including four known (EbpA, CS-4, MJ14, and CHG19) and four novel (HNP-I – HNP-IV) genotypes were identified. Using phylogenetic analysis, genotypes EbpA, CS4, CHG19, HNP-III, and HNP-IV were clustered into zoonotic Group 1, while the remaining three genotypes (MJ14, HNP-I, and HNP-II) clustered into Group 10. The high prevalence of zoonotic genotypes of *E. bieneusi* among pigs suggests that pig farming is a potential source of human infection. Additionally, this is the first identification of genotypes in Group 10 in pigs indicating unique epidemic features of *E. bieneusi* in pigs in Hainan Province, the southernmost part of China.

## Abstract (fr)

*Enterocytozoon bieneusi* est un pathogène fongique zoonotique avec une grande diversité d’hôte qui peut parasiter de nombreux animaux, y compris les humains. Les porcs peuvent jouer un rôle important dans l’épidémiologie d’*E. bieneusi* en tant qu’hôtes réservoirs. Néanmoins, les génotypes d’*E. bieneusi* chez le porc en Chine restent mal connus. Le but de cette étude était de déterminer la prévalence de l’infection par *E. bieneusi* chez les porcs élevés dans des fermes de quatre villes de la province de Hainan, en utilisant la réaction en chaîne par polymérase emboîtée (PCR) de la petite sous-unité partielle du gène de l’ARN ribosomal et de identifier les génotypes des isolats d’*E. bieneusi* sur la base d’une analyse de séquence de la région des espaceurs internes transcrits ribosomiques (ITS). Sur 188 échantillons de selles, *E. bieneusi* a été détecté dans 46,8 % (88/188). Huit génotypes, dont quatre génotypes connus (EbpA, CS-4, MJ14 et CHG19) et quatre génotypes nouveaux (HNP-I à IV), ont été identifiés. Dans une analyse phylogénétique, les génotypes EbpA, CS4, CHG19, HNP-III et HNP-IV étaient regroupés dans le groupe zoonotique 1, tandis que les trois génotypes restants (MJ14, HNP-I et HNP-II) étaient regroupés dans le groupe 10. La prévalence élevée des génotypes zoonotiques d’*E. bieneusi* chez les porcs suggère que l’élevage porcin est une source potentielle d’infection humaine. De plus, il s’agit de la première identification de génotypes du groupe 10 chez les porcs, indiquant des caractéristiques épidémiques uniques d’*E. bieneusi* chez les porcs dans la province de Hainan, la partie la plus méridionale de la Chine.

## Introduction

Microsporidia are a diverse group of obligate intracellular eukaryotic fungi pathogens. They are comprised of approximately 200 genera and 1500 species. To date, 17 species of microsporidia have been recognized as emerging human pathogens [23]. Among them, *Enterocytozoon bieneusi* is the most common species detected both in healthy and immunocompromized individuals that can cause increased mortality through life-threatening diarrhea, particularly in acquired immunodeficiency syndrome (AIDS) patients, children, and transplant recipients [27]. *Enterocytozoon bieneusi* has a high degree of host diversity and can parasitize almost all animal phyla [41]. Thus any animal can act as a potential reservoir host and contribute to environmental pollution and the continuous transmission of the disease [9]. An important step is to adequately control cross-species transmission of *E. bieneusi* by tracing the sources of contamination and elucidating transmission routes. However, the contribution of each animal source to human infections is poorly understood.

Sequence analyses of the internal transcribed spacer (ITS) regions of the rRNA gene have been widely applied for the detection of *E. bieneusi*, and this method is becoming the standard tool for *E. bieneusi* typing [40]. To date, ITS genotyping has contributed to the identification of over 500 genotypes, with 142 genotypes found in humans and 49 genotypes identified both in humans and animals [10, 20, 23, 31, 50, 55]. These recognized genotypes have been divided into 11 phylogenetic groups (Groups 1–11) for phylogenetic analysis [23]. To date, 132 (93.0%) out of the 142 human pathogenic genotypes and 95.9% (47/49) of the zoonotic genotypes belong to Group 1 or Group 2, highlighting the genotypes with public health significance and the nature of cross-species transmission [10, 20, 23, 31, 50, 55]. However, genotypes in Groups 3–11 appear more commonly subject to host adaptation [23]. The contribution of each animal source to human infections can be clarified by the genotyping of *E. bieneusi* in different animals.

Pigs are one of the most important reservoir hosts for *E. bieneusi*. Currently, more than 30 studies on *E. bieneusi* in pigs have been published from 14 countries, and 134 ITS genotypes of *E. bieneusi* have been identified in pigs or wild boars worldwide (Table 1). Among them, 19 genotypes (CHN1, Bfrmr2, CAF1, CS-1, CS-4, D, EbpA, EbpC, EbpD, H, Henan-III, Henan-IV, I, LW1, O, PigEBITS5, PigEBITS7, PigEB10, SH8) have been identified in humans. All genotypes found in the pigs belong to Group 1 (94.8%, 127/134) or Group 2 (5.2%, 7/134), suggesting that pigs play an important role in the epidemiology of *E. bieneusi* as a reservoir host (data based on Ref. [23]). However, the genotypes of *E. bieneusi* in pigs in China are not fully understood and there is a lack of data in many provinces, including Hainan Province (the southernmost part of the country).

**Table 1:** Prevalence and genotype distribution of *Enterocytozoon bieneusi* isolates in pigs and wild boars worldwide.

Continents and countries	Hosts	% (positive/total)	Genotypes (no.)	Ref.
Americas				
Brazil	Pigs	59.3 (54/91)	**EbpA (7), O (3), PigEb10 (1), H (1)**, PigEb2 (16), PigEb4 (16), CS-1 (7), PigEb1 (5), PigEb6 (2), PigEb3, PigEb5, PigEb7 – PigEb9, and PigEb11 – PigEb17 (1 each)	[8]
Peru	Pigs	100.0 (3/3)^a^	**EbpC (3)**	[7]
USA	Pigs	31.7 (64/202)	**D, F, PigEBITS5, PigEBITS7, PigEBITS9** and PigEBITS1 – PigEBITS4, PigEBITS6 and PigEBITS8 (17)	[4]
Asia				
China	Pigs	47.3 (2787/5887)	**EbpC (1140), EbpA(440), CS-4 (58), PigEBITS5 (53), O (51), D(47), Henan-IV (43), H (40), LW1 (17), CS-1 (17), CHS5 (15), CM8 (11), EbpD (7), CHN1 (4), KIN-1 (3), Henan-III (2), I (2), Henan-I (1), CHG23 (1), CM6 (1),** SZZD1 (81), SLTC2 (59), CHG19 (57), SYLA5 (56), EbpB (41), CHC5 (37), SLTC3 (15), PigEB4 (15), CHN7 (14), G (13), CM11 (10), CS-9 (9), SZZA2 (8), HN-1 (6), CS-8 (5), CS-7 (4), XZP-II (4), CS-3 (3), FJF (3), SZZC1 (3), WildBoar8 (3), CHN10, CS-6, HLJ-I, CC-1, SLTC1, SYLA1, SHZA1, HN-2, and XJP-II (2 each), CHN8, CHN9, CS-2, CS-5, CS-10, CHN-RR2, CHG7, FJS, PigITS, PigEBITS3, HLJ-II, HLJ-III, HLJ-IV, SYLA2, SYLA2, SMXBB1, SMXC1, SZZB1, SZZA1, SYLA3, SMXD1, SYLA4, SYLD1, CHG3, SZZD2, SHZC1, SMXD2, SYLC1, HN-3, HN-4, ZJ1, ZJ2, YN1-3, GD1, XZP-I, SCT01, SCT02, and XJP-III (1 each)	[18, 19, 22, 24–26, 46, 48, 49, 53, 56, 57, 62]
	Wild boars	41.2 (147/357)	**EbpC (85), EbpA (22), SH8 (6), D (1), LW1 (1), PigEBITS5 (1),** PigEBITS4 (11), CHC5 (10), WildBoar8 (7), PigEBITS1, SC02, and WildBoar11 (1 each)	[21]
Japan	Pigs	33.3 (10/30)	**H (4), EbpC (2), D, EbpA, and PigEBITS5 (1 each),** Mixed/unknown (1)	[1]
Korea	Pigs	14.2 (67/472)	**PigEBITS5 (4), CAF1 (1), H (1),** PigEBITS3 (2), and PigEBITS4 (2)	[12]
Thailand	Pigs	22.4 (176/787)	**O (38), E (36), EbpC (20), H (8), F (8), LW1 (6), D (5), EbpA (1),** WildBoar5 (9), TMP1^b^ (3), TMP6^b^ (2), PigAYE1 (2), TMP2^b^ – TMP5^b^, TMP7^b^ – TMP11^b^, PigAYE2-3, CS-10, and SHZC1 (1 each)	[17, 30, 43, 44]
Europe				
Austria	Wild boar	13.6 (6/44)	**Henan-I (3), EbpC (2), D (1)**	[29]
Czech Republic	Pigs	93.7 (74/79)	**F (70), D (2), Peru9 (2)**	[35]
	Wild boar	8.7 (20/231)	**EpbA (10), D (5), EbpC (1),** WildBoar4 (2), G (1), and WildBoar3 (1)	[29]
Germany	Pigs	23.3 (21/90)	**F (7), H (2), O (4), E (1), Bfrmr2 (1),** G (2), E1, F1, and Bfrmr5 (1 each)	[6, 33, 34]
Poland	Wild boar	7.6 (10/129)	**EbpC (3), EbpA (2),** WildBoar5 (2), WildBoar2, WildBoar3, and WildBoar6 (1 each)	[29]
Slovak Republic	Wild boar	3.6 (2/56)	**D (1),** WildBoar1 (1)	[29]
Spain	Pigs	20.6 (7/34)	**I (1)**	[9]
Switzerland	Pigs	25.7 (28/109)	**EbpA (12), EbpC (7), EbpD (3),** EbpB (6)	[3]

The genotypes found in humans previously are shown in bold.

a Genotyping study using confirmed *E. bieneusi*-positive isolates.

b Invalid genotypes as the sequences submitted to GenBank contain an incomplete ITS region.

In China, the pig industry is a major economic component in which humans and pigs live in crowded conditions, and the disease can be easily spread with a potentially major impact on the economy [57]. Hainan Province is a relatively isolated island, where *E. bieneusi* is prevalent in multiple animals including farmed and wild animals [60, 61]; moreover, it has been found in humans with diarrhea (unpublished data). The pig industry represents an important poverty alleviation project in Hainan Province. However, information on the prevalence and genetic characteristics of *E. bieneusi* in pigs in this province is lacking. The aims of this study were to determine the prevalence and genotype distribution of *E. bieneusi* in pigs from four cities of Hainan Province, to provide useful information to assess the risk of zoonotic transmission.

## Materials and methods

### Ethics statement

Before beginning work on the present study, we contacted the farm owners and obtained their permission to have their animals involved. Written informed consent was obtained from the owners for the participation of their animals in this study. The protocol was also reviewed and approved by the Ethics Committee of Hainan Medical University.

### Collection of fecal specimens

**Figure 1:**
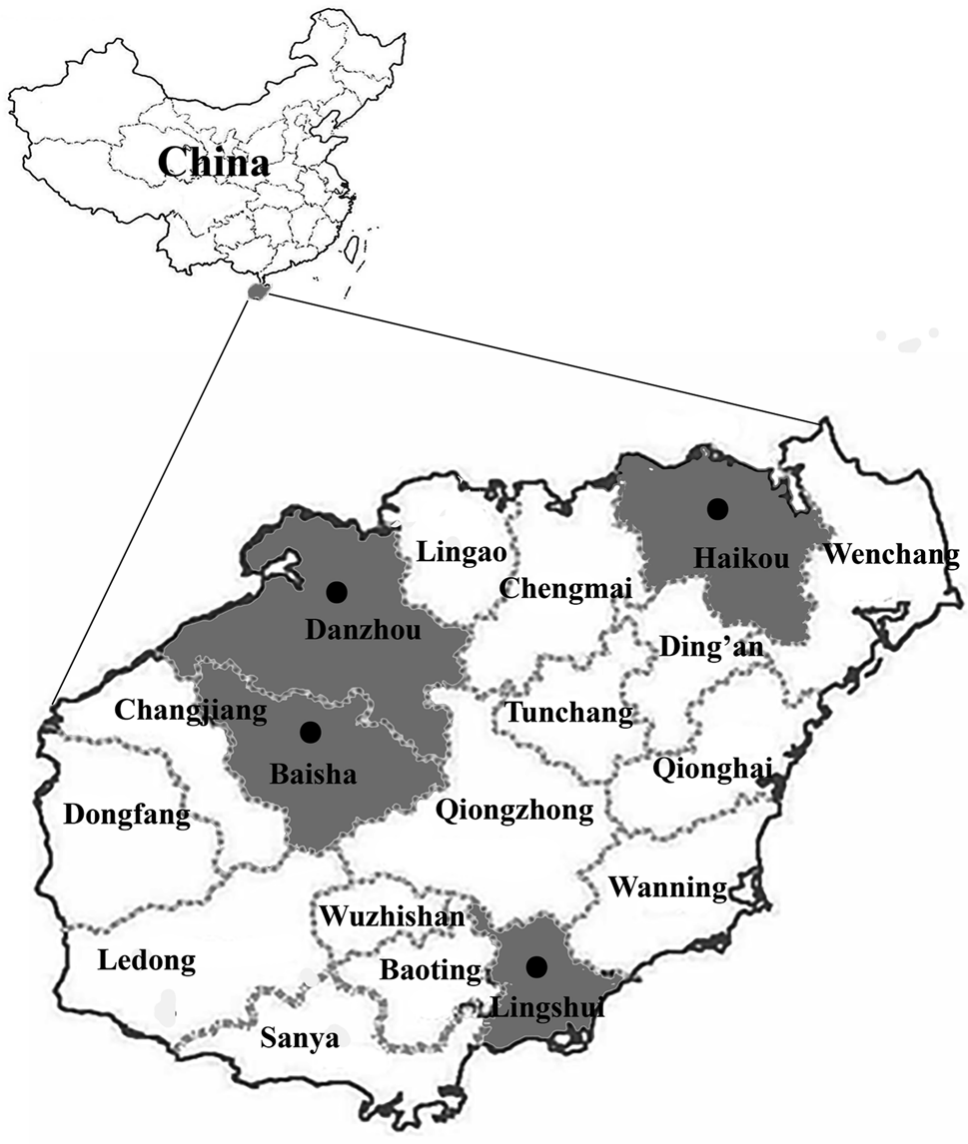
Specific locations where samples were collected in this study. Dots indicate sampling points.

From March to June 2019, a total of 188 fresh fecal samples were collected from four pig farms in four cities of Hainan Province, including Baisha (*n* = 30), Danzhou (*n* = 58), Haikou (*n* = 20), and Lingshui (*n* = 80) (Fig. 1, Table 2). Farms were selected based on the owners’ willingness to participate and the accessibility of animals for sampling. The number of collected specimens accounted for 20–30% of total pigs on each farm. All fecal specimens were collected from the ground immediately after defecation using a sterile disposable latex glove and placed in individual labeled sterile tubes. The ages of the pigs sampled in this study belonged to two groups: one group containing 61 pre-weaned and post-weaned pigs aged ≤ 60 days, and the other group containing 127 fattening pigs aged more than 60 days. At the time of sampling, all pigs were in good health. All bags were transported to our laboratory in a cooler with ice packs within 24 h, and stored at 4 °C until processing (within 48 h).

**Table 2:** Prevalence and genotype distribution of *E. bieneusi* isolates in farm-raised pigs in Hainan Province.

Category	Positive/examined (%)	Genotype/s (no.)	% zoonotic genotypes
Location			
Baisha	16/30 (53.3)	MJ14 (12), CS-4 (2), HNP-I (1), HNP-II (1)	12.5
Danzhou	20/58 (34.5)	CS-4 (10), CHG19 (9), HNP-III (1)	50.0
Haikou	11/20 (55.0)	CS-4 (10), EbpA (1)	100.0
Lingshui	41/80 (51.3)	CS-4 (37), EbpA (3), HNP-IV (1)	97.6
Age			
≤60 d	36/61 (54.5)	CS-4 (28), CHG19 (4), EbpA (4)	88.9
>60 d	52/127 (40.9)	CS-4 (31), MJ14 (12), CHG19 (5), HNP-I – HNP -IV (1 each)	59.6
Total	88/188 (46.8)	CS-4 (59), MJ14 (12), CHG19 (9), EbpA (4), HNP-I – HNP-IV (1 each)	71.6

### DNA extraction

All fecal specimens of the pigs were collected and sieved through an 8.0-cm-diameter sieve with a pore size of 45 μm. Filtrates were concentrated by centrifugation at 1500× *g* for 10 min. Approximately 200 mg of each processed sample was homogenized in 1400 μL of DNA extraction buffer ASL. Genomic DNA of each specimen was extracted using a QIAamp DNA stool mini kit (QIAgen, Hilden, Germany), according to the manufacturer’s recommendations. Extracted DNA was used for PCR analysis. All samples were stored at −20 °C in a refrigerator.

### PCR amplification

All DNA preparations were analyzed for the presence of *E. bieneusi* by nested PCR amplification of the ITS region of the rRNA gene with a set of specific primers. Primers and cycle parameters were designed by Mirjalal [28]. TaKaRa Taq DNA Polymerase (TaKaRa Bio Inc., Tokyo, Japan) was used for all PCR amplifications at a final volume of 25 μL. A positive control with rat-derived genotype Peru 8 DNA and a negative control with no added DNA were amplified in all PCR tests. All secondary PCR products were subjected to electrophoresis on 1.5% agarose gels and visualized by staining with GelRed (Biotium Inc., Hayward, CA, USA).

### Nucleotide sequencing and analyzing

All secondary PCR products positive for *E. bieneusi* were sequenced by Sangon Biotech Co., Ltd. (Shanghai, China). Sequence accuracy was confirmed by two-directional sequencing and by sequencing additional PCR products, as required. The genotypes of *E. bieneusi* were identified through the comparison of nucleotide sequences obtained with each other, and from published GenBank sequences using the Basic Local Alignment Search Tool (BLAST) (http://blast.ncbi.nlm.nih.gov/Blast.cgi) and ClustalX 1.83 (http://www.clustal.org/). The obtained genotypes of *E. bieneusi* were given a published name when identical to the genotypes in GenBank [40]. In parallel, the genotypes that produced ITS sequences with any single nucleotide substitutions, deletions, or insertions were confirmed by DNA sequencing of at least two PCR products and considered as novel genotypes. All were given a genotype name through the addition of roman numerals behind the abbreviation HNP (Hainan Pig), according to their order of appearance. All genotypes were named based on a 243 bp sequence of the ITS gene region of *E. bieneusi*, according to the established nomenclature system [40].

### Phylogenetic analysis

To confirm the genogroup designation and to assess the genetic relationships of obtained novel ITS genotypes of *E. bieneusi* with known genotypes, a phylogenetic analysis was performed through the construction of a neighbor-joining tree using the program Mega X (http://www.megasoftware.net/) based on the evolutionary distances calculated by the Kimura-2-parameter model. The reliability of these trees was assessed using bootstrap analysis with 1000 replicates.

### Statistical analysis

Differences in the infection rates among different locations and ages were assessed using a Chi-square test with SPSS Version 22.0 software (IBM Corp., Armonk, NY, USA). *P-*values < 0.05 were considered significant.

### Nucleotide sequence accession numbers

Representative nucleotide sequences obtained in the study were deposited in the GenBank database under accession numbers MN630620–MN630623.

## Results

### Occurrence of *E. bieneusi* in pigs

*E. bieneusi* was detected in 46.8% (88/188) of the pig samples. The pathogen was detected in all four cities in Hainan Province, with peak infection rates of 55.0% (11/20) in Haikou, followed by Baisha (53.3%; 16/30), Lingshui (51.3%; 41/80), and Danzhou (34.5%, 20/58) (Table 2). There were no significant differences in the prevalence among different locations (*χ* ^2^ = 2.614, *p* > 0.05). Regarding the two age groups, the infection rate in the younger group (aged ≤ 60 days) was 54.5% (36/61), which was significantly higher than that in the older group (aged > 60 days) at 40.9% (52/127) (*χ* ^2^ = 5.405, *p* < 0.05) (Table 2).

### Genetic characterizations and genotypic distribution of *E. bieneusi* in pigs

Through sequence analysis of 88 *E. bieneusi* isolates, eight ITS genotypes were identified with a total of 16 polymorphic sites observed among them (Table 3). Four known genotypes (CS-4, MJ14, CHG19, and EbpA) and four novel genotypes termed HNP-I – HNP-IV were identified (Table 2). Among the genotypes, CS-4 was the most prevalent and identified in 59 (59/88, 67.0%) positive specimens, followed by MJ14, CHG19, and EbpA found in 12 (12/88, 13.6%), 9 (8/88, 10.2%), and 4 (4/88, 4.5%) specimens, respectively. Genotypes HNP-I – HNP-IV were identified in one specimen each (Table 2).

**Table 3:** Variation of the ITS gene sequences of *E. bieneusi* isolates in farm-raised pigs in Hainan Province.

Genotypes	GenBank accession no.	Nucleotide at position															
		2	11	14	31	32	82	96	132	137	138	142	144	148	159	197	224
Known																	
CS-4	MK778898	–	G	G	T	G	C	G	G	G	C	C	A	G	T	A	C
CHG19	MH817463	–	G	G	T	A	C	T	G	G	C	C	A	G	T	A	C
EbpA	AF076040	–	G	G	T	A	T	T	G	A	C	T	G	G	T	G	C
MJ14	MK348513	–	A	G	C	A	T	G	C	G	T	T	A	G	G	A	C
Novel																	
HNP-I	MN630621	C	A	G	C	A	T	G	C	G	T	T	A	G	G	A	C
HNP-II	MN630622	–	A	G	C	A	T	G	C	G	T	T	A	A	G	A	C
HNP-III	MN630620	–	G	A	T	A	C	T	G	G	C	C	A	G	T	A	C
HNP-IV	MN630623	–	G	G	T	G	C	G	G	G	C	C	A	G	T	A	T

Genotypes HNP-I (MN630621) and HNP-II (MN630622) had the largest similarities with genotype MJ14 (MK348513), while genotype HNP-I (MN630621) had a single nucleotide (C) insertion at position 2. Genotype HNP-II (MN630622) had one base variation at position 148 (G → A). In contrast, genotypes HNP-III (MN630620) and HNP-IV (MN630623) had one base difference compared to genotypes CHG19 (MH817463) and CS-4 (MK778898) at positions 13 (G → A) and 223 (C → T), respectively.

Genotype CS-4 was identified in all four of the locations with EbpA in Haikou and Lingshui, MJ14, HNP-I, and HNP-II in Baisha, CHG19 and HNP-III in Danzhou, and HNP-IV in Lingshui, respectively. Considering the two age groups, genotypes CS-4 and CHG19 were found in both age groups, while genotype EbpA was exclusively found in the younger group. Genotypes HNP-I – HNP-IV and MJ14 were exclusive to the older groups (Table 2).

### Phylogenetic relationship of *E. bieneusi* genotypes

**Figure 2:**
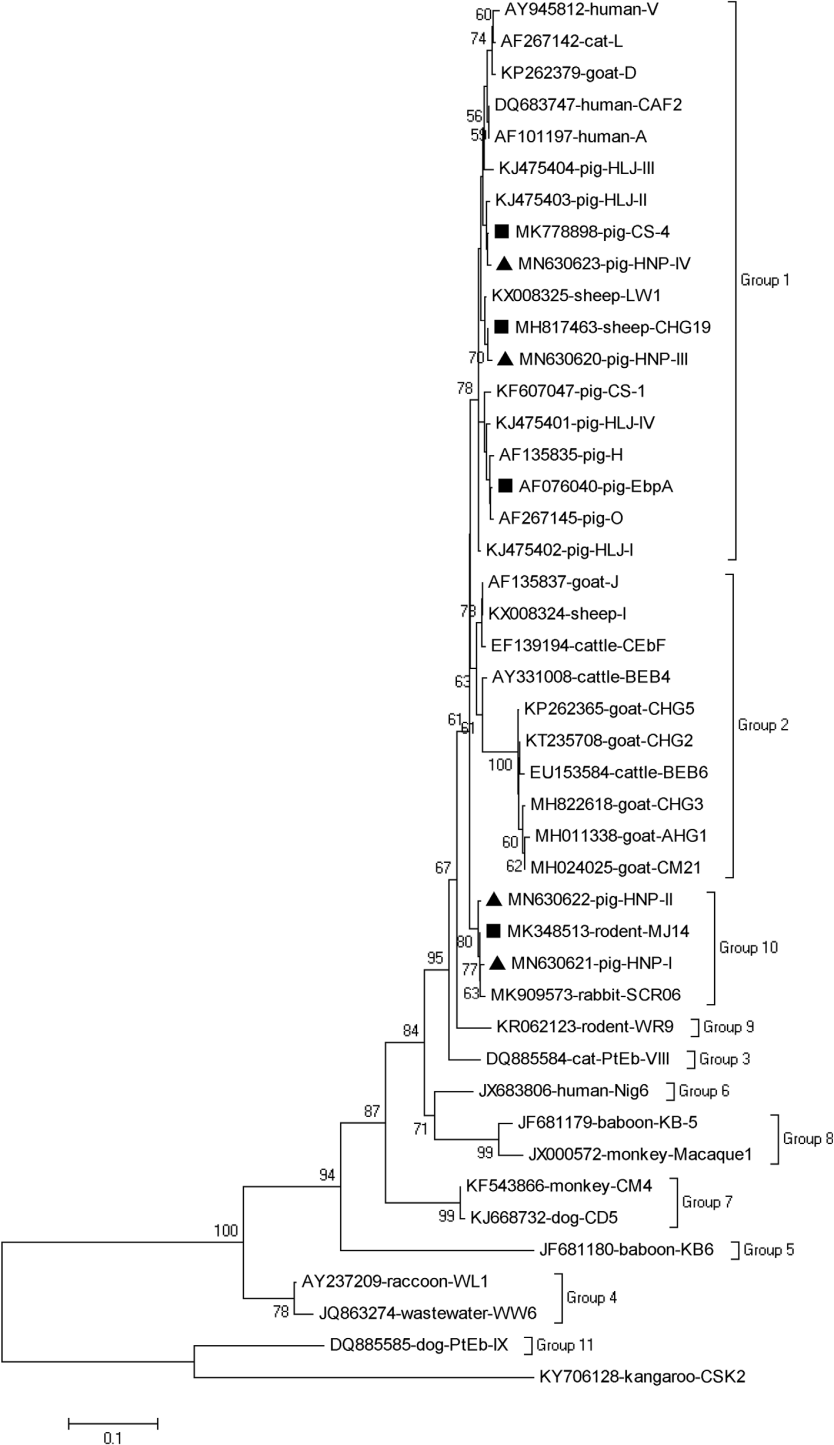
Phylogenetic tree based on the neighbor-joining analysis of ITS sequences. Phylogenetic relationships of *E. bieneusi* genotypes identified in pigs here and other known genotypes deposited in GenBank were inferred by a neighbor-joining analysis of ITS sequences based on genetic distance by the Kimura-2-parameter model. The numbers on the branches are percent bootstrapping values from 1000 replicates. Each sequence is identified by its accession number, host origin, and genotype designation. The *E. bieneusi* genotype CSK2 (KY706128) from white kangaroo was used as the outgroup. The squares and triangles filled in black indicate novel and known genotypes identified in this study, respectively.

Based on the phylogenetic analysis of the neighbor-joining tree of the ITS gene sequences of *E. bieneusi*, genotypes CS-4, CHG19, EbpA, HNP-III, and HNP-IV were located in zoonotic Group 1, while genotypes MJ14, HNP-I, and HNP-II were clustered into Group 10 (Fig. 2).

## Discussion

In this study, the overall infection rate of *E. bieneusi* in pigs was 46.8% (88/188), which was similar to that reported in other provinces of China, such as Heilongjiang (45.3%; 39/86) [22], Henan (45.5%; 408/897) [48], Tibet (43.2%; 309/715) [18], and Xinjiang (48.6%; 389/801) [19]. A total of 30 studies on *E. bieneusi* in pigs or wild boars from 14 countries have been reported with average infection rates of 42.3% (3291/7784) in pigs and 22.6% (185/817) in wild boars. Among them, 14 of the studies are from China with an average infection rate of 47.0% (2934/6244), ranging from 10.2% (5/49) in Heilongjiang to 100% (2/2) in Inner Mongolia. The prevalence of *E. bieneusi* in pigs from developed countries has been reported at rates ranging from 3.6% (2/56) to 93.7% (74/79), which is lower than rates reported in developing countries (10.2–100.0%).

As for other opportunistic pathogens, the prevalence of *E. bieneusi* is closely associated with host age. In the present study, younger pigs (54.5%; 36/61) had significantly higher infection rates of *E. bieneusi* than older pigs (40.9%; 52/127) (*χ* ^2^ = 5.405, *P* < 0.05), in agreement with previous studies. In studies from Thailand, the prevalence of *E. bieneusi* in pigs aged 2–3.9 months was significantly higher than other age groups [17]. Likewise, Li et al. reported that among the three age groups, the nursery group had the highest prevalence rates of *E. bieneusi* compared to those in the pre-weaned and growing groups [22]. Meanwhile, Zhao et al. showed that post-weaned piglets aged 2–3 months presented high infection rates (89.5%) [57]. The prevalence of *E. bieneusi* in young pigs was higher than that of adults, most likely due to the underdeveloped immune systems of the young animals.

In the present study, eight genotypes of *E. bieneusi* were identified with four previously reported genotypes CS-4, MJ14, CHG19, and EbpA, and four novel genotypes HNP-I – HNP-IV. Among them, genotype CS-4 was dominant with the highest occurrence (67.0%; 59/88) and the widest distribution (detected in all four investigated areas) in pigs from Hainan. This genotype has also been reported in other animals, including non-human primates [14], sheep [13], and horses [32]. It was shown that genotype CS-4 has the ability to infect humans, particularly children [51], and has been found in river water [11]. Genotype EbpA was identified in four pigs in this study. A large variety of animals have been reported to be infected with genotype EbpA. In addition to pigs, EbpA has been detected in non-human primates [37, 39], cattle [5, 6, 11, 58], sheep [42], goats [42, 59], deer [54], horses [32, 45], house mice [36], and birds [15, 16], with a wide host range. This genotype has also been found in humans from the Czech Republic [38], Nigeria [2], and China [47], highlighting its zoonotic nature. Recently, Li et al. reported that genotype EbpA is present on the surfaces of vegetables and fruits, highlighting a possible risk of foodborne-related disease outbreaks [23].

The other two known genotypes (MJ14 and CHG19) identified in this study have only been found in animals to date. To our knowledge, genotype MJ14 (MK348513) was originally identified and designated in binturong (*Arctictis binturong*) from Yunnan Province (unpublished). It was also termed HNR-VI (MN267057) and found in Asiatic brush-tailed porcupines from Hainan Province (unpublished). The identification of genotype MJ14 (HNR-VI) in pigs and rodents in the same province not only suggests that the genotype has an extensive host range, but that potential cross-species transmission between pigs and rodents can occur. Genotype CHG19 has been identified in pigs from Henan [48] and Shaanxi [49], in captive Eurasian wild boars from Sichuan [21], in goats from Yunnan [42], in horses from Xinjiang [32] and on the surfaces of vegetables and fruits from Henan [23]. This suggests that CHG19 has the ability to infect a large variety of animals. Despite the lack of detection of CHG19 in humans, it should continue to be monitored.

Four novel genotypes were identified in this study. The number of ITS genotypes of *E. bieneusi* is increasing dramatically with a larger number of isolates of *E. bieneusi* sequenced. It is conceivable that if sufficient isolates are sequenced, all nucleotides in the ITS could be polymorphic [4]. Meanwhile, although the ITS region is 243 bp in length for the majority of *E. bieneusi* genotypes, length variations of the ITS region of rDNA of *E. bieneusi* have been identified in five genotypes, with one or two nucleotide deletions (241 bp or 242 bp) [52, 53], and three genotypes with a single nucleotide insertion (244 bp) in the ITS region [52, 60]. It was observed that there were 244 bp in the ITS region of genotype HNP-I. Other genetic markers are required to substantiate these observations.

Currently, 95.5% (128/134) of the genotypes identified in pigs or wild boars were clustered into Group 1, while the other six genotypes (I, CHN1, CHN9, CHN10, SZZD2, and CHG3) clustered into Group 2 (Table 1). Genotypes CS-4, CHG19, and EbpA, and two novel genotypes (HNP-III and HNP-IV) were located in zoonotic Group 1, while genotype MJ14 (MK348513) and the other two novel genotypes (HNP-I and HNP-II) were clustered into Group 10.

## Conclusions

This is the first study to report the identification of *E. bieneusi* in pigs in Hainan Province, with a high prevalence and wide occurrence demonstrated (detected in all four investigated areas). The findings that the two known human-pathogenic genotypes (CS-4 and EbpA) are in high proportions, and that genotype CHG19 as well as two novel genotypes (HNP-III and HNP-IV) of *E. bieneusi* belong to zoonotic Group 1, indicate the possibility of transmission between pigs and humans. This study represents the first identification of genotypes in Group 10 (MJ14, HNP-I, and HNP-II) in pigs, indicating the unique epidemic features of *E. bieneusi* in pigs in Hainan Province.
